# Draft Whole-Genome Sequence of Urease-Producing *Sporosarcina koreensis*

**DOI:** 10.1128/genomeA.00096-16

**Published:** 2016-03-17

**Authors:** Sara Abdul Majid, Michael F. Graw, Hanh Nguyen, Anthony G. Hay

**Affiliations:** a Department of Research, Weill Cornell Medical College in Qatar, Doha, Qatar; b Department of Microbiology, Cornell University, Ithaca, New York, USA

## Abstract

Urease-producing microbes are of significance due to their potential application in biocement production. *Sporosarcina koreensis* Q1 is a urease-producing bacterium belonging to the phylum *Firmicutes*. Here, we present the draft whole-genome sequence of *S. koreensis* Q1, isolated from a barchan sand dune in Qatar.

## GENOME ANNOUNCEMENT

Urease-producing microbes have been extensively studied for their potential application in biocement production for soil stabilization (1), aquaculture (2), and engineering works (3, 4). Biocementation is a technology relying on microbiologically induced calcite precipitation (MICP) via pH changes brought on by the hydrolysis of urea and release of ammonium by urease-producing microorganisms. *Sporosarcina* spp. are heterotrophic, Gram-positive bacteria that belong to the *Planococcaceae* family, *Bacilli* order, of the phylum *Firmicutes*. There are 16 species identified to date (http://www.bacterio.net) (5). A number of these species have been documented as urease-producers including *S. ginsengisoli* (6) and *S. pasteurii* (7). A urease-producing strain of *Sporosarcina pasteurii* was previously sequenced and published in Genome Announcements (8), but the sequence was removed from NCBI due to contamination and is no longer accessible (http://www.ncbi.nlm.nih.gov/nuccore/AYOX00000000.1).

Strain Q1 was isolated from the sand of a barchan dune located south of Doha city in the State of Qatar (25.009450°, 51.340490°) in April of 2013. The sand was used as an inoculum in nitrogen free minimal medium supplemented with glucose and urea as sole carbon and nitrogen sources, respectively. The strain produced urease activity on urease indicator medium and was able to form water-stable aggregates (>0.5 mm) when mixed with fine sand in the presence of calcium and urea.

Initial characterization was done via partial 16S rRNA gene sequencing; the strain was found to be 98% identical to *Sporosarcina koreensis* strain F73. Library construction was performed using the Nextera XT kit (Illumina, USA). After library construction, the sample was spiked with 50% PhiX and sequenced on an Illumina HiSeq2500 instrument using 250 bp reads according to the manufacturer’s protocol at the Center of Biotechnology in Cornell University, Ithaca, New York.

The genome was assembled with Velvet (9) using the Velvet Optimizer add-on. The final assembly used a k-mer length of 157 and a coverage cutoff of 5.53. The assembly produced 4,320,341 bp of sequence across 426 contigs with an *N*_50_ of 72,066 bp, a longest sequence of 212,353 bp, and a G+C content of 44.5%. A total of 51 putative rRNA genes were identified using the BLASTn_RNA script within WebMGA (10); 10 of these resulted in highly significant (e-values <10^−100^), high identity (99% similarity) alignments against *Sporosarcina luteola* (NR114283.1)*, saromensis* (NR114249.1)*,* and *koreensis* (NR043526.1) partial 16S rRNA genes within the NCBI 16S rRNA database. The draft genome was annotated using the Rapid Annotations using Subsystem Technology (RAST) v2.0. RAST identified 4,413 coding sequences spanning 439 subsystems, dominated by the following features: amino acids and derivatives (14%), carbohydrates (9%), and cofactors, vitamins, prosthetic groups, and pigments (9%). One of the subsystems includes the seven urease genes (3 structural *UreaA, UreaB, UreaC,* and 4 accessory *UreaD, UreaE, UreaF, UreaG*). We anticipate that this genome sequence will be of value to those studying MICP and/or related *Sporosarcina* species.

### Nucleotide sequence accession numbers.

This whole-genome shotgun project has been deposited in DDBJ/EMBL/GenBank under the accession no. LQYA00000000. The version described in this paper is the first version, LQYA01000000.
