# Erratum for Abdul Majid et al., Draft Whole-Genome Sequence of Urease-Producing *Sporosarcina koreensis*

**DOI:** 10.1128/genomeA.00507-16

**Published:** 2016-05-26

**Authors:** Sara Abdul Majid, Michael F. Graw, Hanh Nguyen, Anthony G. Hay

**Affiliations:** a Department of Research, Weill Cornell Medical College in Qatar, Doha, Qatar; b Department of Microbiology, Cornell University, Ithaca, New York, USA

## ERRATUM

Volume 4, no. 2, e00096-16, 2016. Page 1: In the Funding Information section, grant number “09-546-2-206” should read “6-059-2-023.”

